# L1CAM protein expression is associated with poor prognosis in non-small cell lung cancer

**DOI:** 10.1186/1476-4598-10-127

**Published:** 2011-10-10

**Authors:** Verena Tischler, Marco Pfeifer, Silke Hausladen, Uwe Schirmer, Anne-Katrine Bonde, Glen Kristiansen, Martin L Sos, Walter Weder, Holger Moch, Peter Altevogt, Alex Soltermann

**Affiliations:** 1 Institute of Surgical Pathology, University Hospital Zurich, Zurich, Switzerland; 2 Tumour Immunology Programme D015, German Cancer Research Center, Heidelberg, Germany; 3 Institute of Molecular Cancer Research, University of Zurich, Zurich, Switzerland; 4 Max Planck Institute for Neurological Research with Klaus-Joachim-Zülch Laboratories of the Max Planck Society and the Medical Faculty of the University of Köln, Köln, Germany; 5 Clinic of Thoracic Surgery, University Hospital Zurich, Zurich, Switzerland

**Keywords:** L1 cell adhesion molecule, epithelial-mesenchymal transition, tumor-stroma interface, prognostic marker, non-small cell lung cancer, tissue microarray

## Abstract

**Background:**

The L1 cell adhesion molecule (L1CAM) is potentially involved in epithelial-mesenchymal transition (EMT). EMT marker expression is of prognostic significance in non-small cell lung cancer (NSCLC). The relevance of L1CAM for NSCLC is unclear. We investigated the protein expression of L1CAM in a cohort of NSCLC patients. L1CAM protein expression was correlated with clinico-pathological parameters including survival and markers of epithelial-mesenchymal transition.

**Results:**

L1CAM protein expression was found in 25% of squamous cell carcinomas and 24% of adenocarcinomas and correlated with blood vessel invasion and metastasis (p < 0.05). L1CAM was an independent predictor of survival in a multivariate analysis including pT, pN, and pM category, and tumor differentiation grade. L1CAM expression positively correlated with vimentin, beta-catenin, and slug, but inversely with E-cadherin (all p-values < 0.05). E-cadherin expression was higher in the tumor center than in the tumor periphery, whereas L1CAM and vimentin were expressed at the tumor-stroma interface. In L1CAM-negative A549 cells the L1CAM expression was upregulated and matrigel invasion was increased after stimulation with TGF-beta1. In L1CAM-positive SK-LU-1 and SK-LC-LL cells matrigel invasion was decreased after L1CAM siRNA knockdown.

**Conclusions:**

A subset of NSCLCs with vessel tropism and increased metastasis aberrantly expresses L1CAM. L1CAM is a novel prognostic marker for NSCLCs that is upregulated by EMT induction and appears to be instrumental for enhanced cell invasion.

## Background

The 220 kDa transmembrane glycoprotein L1CAM belongs to the neuronal immunoglobulin superfamily of cell adhesion molecules and was first described in neural cell migration [1] for review see [2]. L1CAM is comprised of six IgG-like domains and five fibronectin-type III repeats, followed by a transmembrane region and a highly conserved cytoplasmic tail [2,3]. L1CAM protein expression was observed in renal cell cancer, ovarian carcinomas, melanoma, colon cancer, pancreatic cancer, and small cell lung cancer [4-11]. Currently, no data of L1CAM are available for non-small cell lung cancer (NSCLC). Membranous L1CAM enhanced motility of HEK293 cells by interfering with integrin-dependent signaling pathways, inducing endocytosis of beta 1 integrin [12]. L1CAM expression activated extracellular signal-regulated kinase (Erk)-dependent gene regulation and induced NFκB activity conferring increased cell motility and invasion [13,14].

We previously reported the prognostic importance of epithelial-mesenchymal transition (EMT) for lung cancer [15]. EMT is a cell biological program in which cancer cells lose their epithelial features like E-cadherin expression and up-regulate mesenchymal proteins like vimentin or periostin [15]. Recently, L1CAM has been linked to EMT because protein overexpression decreased the junctional expression of E-cadherin and promoted colony scattering in breast carcinoma cells [16]. As a consequence of EMT, cancer cells acquire a fibroblastic phenotype which enables them to detach from their lattice and to become migratory and invasive. The EMT program is inducible by growth factors like transforming growth factor beta1 (TGF-beta1) or hepatocyte growth factor (HGF) [17]. Several intracellular signaling cascades are involved thereafter, including MAPK and AKT pathways (for review see [17-20]). In pancreatic and endometrial carcinoma cells, L1CAM up-regulation was dependent on TGF-beta1 induction of the EMT transcriptional factor slug [9,21]. The zinc finger protein slug binds to the E-box motif of the E-cadherin promoter thereby repressing E-cadherin [22]. Further, L1CAM is a target gene of beta-catenin/TCF (T-cell factor) signaling [23]. Topographically, L1CAM was exclusively detected at the invasive front of colorectal cancer and its knockdown reduced haptotactic motility [23].

The present study aimed to investigate the relationship between L1CAM and indicators of EMT in a large NSCLC patient cohort as well as in NSCLC cell lines. Further, we tested the hypothesis that L1CAM is relevant for patient survival.

## Results and Discussion

### Results

#### L1CAM expression in NSCLC

**Figure 1:**
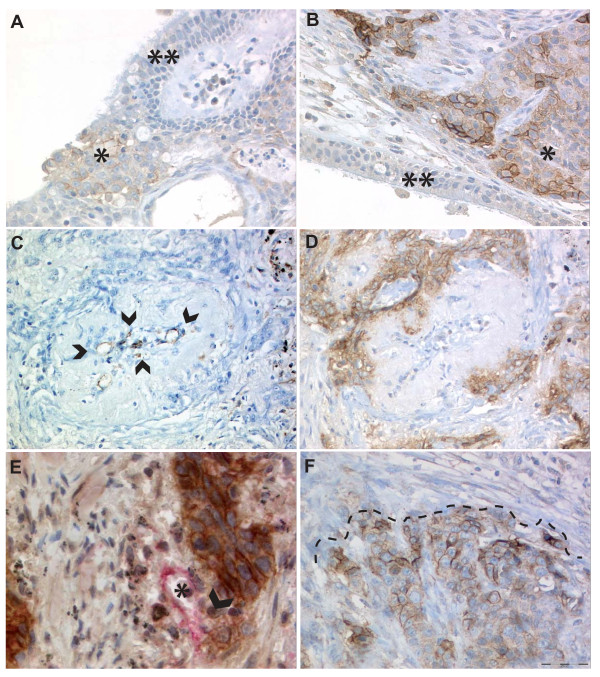
**Expression of L1CAM in NSCLC**. **A/B) **SCC with expression of L1CAM (*) whereas the bronchial ciliated and focally metaplastic epithelium is negative (**), magnification 200x. Note on Figure 1B the pronounced expression of L1CAM in some areas of the tumor-stroma interface. **C/D) **Serial section, **C **CD31 (endothelial cells marked by arrowheads) and **D **L1CAM, of a SCC surrounding and partially invading the media of a blood vessel. Note that intratumoral vessels regularly show profound remodeling of the arteriolar wall with disappearance of the elastic layers normally bordering the media myocytes, magnification 200x. **E) **L1CAM positive tumor cells (brown) destroying the vessel wall lined by CD31 positive (red) endothelial cells. **F) **Accentuated L1CAM expression at the tumor-stroma interface (dashed line) and weaker (heterogeneous) L1CAM expression in the lower part of the picture representing a central part of the tumor. Hematoxylin counterstain was used for all slides.

**Figure 2:**
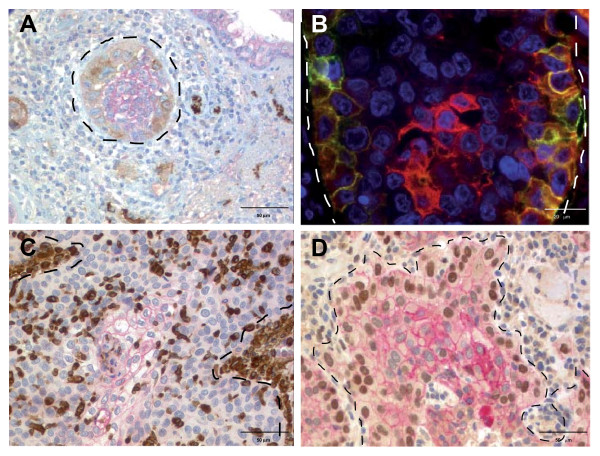
**L1CAM and EMT marker expression patterns at the tumor-stroma interface**. **A) **SCC with expression of L1CAM at the tumor-stroma interface (brown). The tumor center shows moderate E-cadherin expression (red). At the tumor-stroma interface, E-cadherin expression is decreased. Note the strong positivity of small peripheral nerves for L1CAM. **B) **Double IF staining for L1CAM (green) and E-cadherin (red): L1CAM is expressed at the tumor border and E-cadherin expression is strongest in the tumor center. E-cadherin expression is decreased at the tumor border (yellow). **C) **Membranous E-cadherin (red) is expressed in the tumor center and decreased towards the tumor-stroma interface. Two strong Vimentin positive (brown) stromal cell aggregates are marked with dotted lines (upper left and mid to lower right). Note that most of the Vimentin positive cells show nuclear morphology of the tumor cells. CD68 staining to exclude Vimentin positive macrophages was not performed. **D) **Decrease of membranous E-cadherin (red) and strong nuclear expression of slug (brown) at the tumor-stroma interface of a SCC. In the tumor center E-cadherin is strongly but slug is not expressed (blue nuclei). Hematoxylin and DAPI counterstain were used, respectively. Tumor-stroma interfaces are marked by dotted lines.

L1CAM protein expression (any sum intensity > score 0) was found in 25% of the tumors. Similarly, 25% of the SCC and 24% of the ADCA expressed L1CAM (table 1). An overview of L1CAM expression and clinicopathological parameters is shown in table 1. Normal lung tissue, endothelial cells and bronchial epithelium were negative for L1CAM stained on whole sections (Figure 1A, B). L1CAM expression significantly correlated with the pM1 category (p = 0.031) and with blood vessel infiltration (p = 0.036; table 1). Examples of vessel infiltration and blood vessel tropism are shown in Figure 1C-E. L1CAM expression was found to be strongest at the tumor-stroma interface (Figure 1B, F and 2A, B). Expression of L1CAM was found to be heterogeneous in 63/468 (13.5%) of cases which was confirmed in randomly selected cases by whole sections (Figure 1F).

**Table 1:** L1CAM expression and clinicopathological parameters

	n	%	L1CAM negative	%	L1CAM positive	%	p-value/ tau
							

**Total**	468	100	353	75.4	115	24.6	

							

**Age ≤64 years**	227	48.5	178	78.4	49	21.6	ns

**Age > 64 years**	241	51.5	175	72.6	66	27.4	

							

**Male**	325	69.4	243	74.8	82	25.2	ns

**Female**	143	30.6	110	76.9	33	23.1	

							

**SCC**	242	51.7	181	74.8	61	25.2	ns

**ADCA**	226	48.3	172	76.1	54	23.9	

							

**pT1**	98	20.9	78	79.6	20	20.4	ns

**pT2**	256	54.7	195	76.2	61	23.8	

**pT3**	69	14.7	50	72.5	19	27.5	

**pT4**	45	9.6	30	66.7	15	33.3	

							

**pN0**	244	52.1	186	76.2	58	23.8	ns

**pN1**	142	30.3	110	77.5	32	22.5	

**pN2**	72	15.4	51	70.8	21	29.2	

**pN3**	10	2.1	6	60	4	40	

							

**pM0**	430	91.9	330	76.7	100	23.3	*0.031*/

**pM1**	38	8.1	23	60.5	15	39.5	0.1

							

**G1**	28	6	22	78.6	6	21.4	ns

**G2**	245	52.3	187	76.3	58	23.7	

**G3**	195	41.7	144	73.8	51	26.2	

							

**Size < 3.7cm**	241	51.5	189	78.4	52	21.6	ns

**Size ≥ 3.7cm**	227	48.5	164	72.2	63	27.8	

							

**No vessel infiltration**	249	53.2	197	79.1	52	20.9	*0.036*/

**Vessel infiltration**	219	46.8	156	71.2	63	28.8	0.09

Neg. negative, pos. positive, ns not significant, tau correlation coefficient

#### L1CAM expression is correlated with poor overall and disease free survival

**Figure 3:**
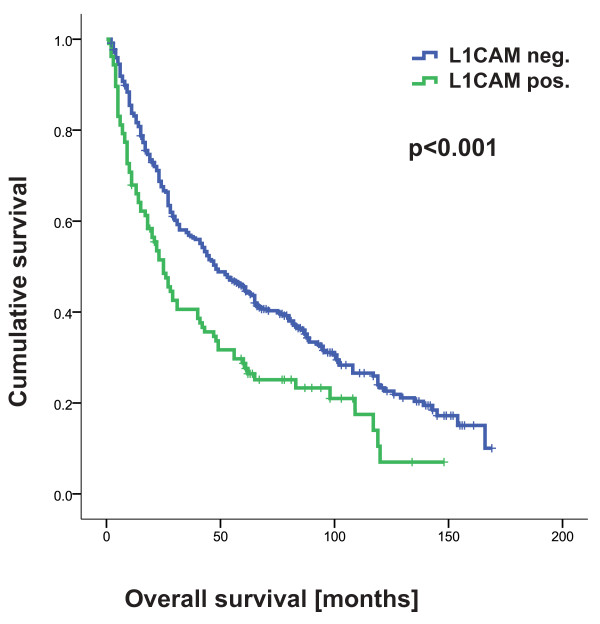
**L1CAM expression is correlated with shortened overall survival**.

In the total cohort, L1CAM expression was associated with unfavourable OS (p < 0.001) and PFS (p < 0.001) in univariate analysis (table 2 for OS see Figure 3). All other EMT markers were not associated with prognosis. The prognostic impact of L1CAM expression was independent of pT, pN, pM and tumor grade in a multivariate analysis (table 3) for both OS and PFS.

**Table 2:** Univariate cumulative survival analysis (54 months)

Survival	L1CAM expression	Cases	Events	Estimate	95% CI lower	95% CI upper	p-value
							

**OS**	negative	346	245	69.32	62.81	75.82	*< 0.001*

	positive	106	83	46.23	36.77	55.69	

							

**PFS**	negative	346	254	61.2	54.46	67.95	*< 0.001*

	positive	106	87	37.43	27.88	46.98	

OS overall survival, PFS progression free survival, CI confidence interval

**Table 3:** Multivariate survival analysis

Parameters	p-value	Exp(B)	95% CI lower	95% CI upper
				

**OS**				

L1CAM	*0.043*	1.31	1.01	1.7

pT	*< 0.001*	1.47	1.29	1.68

pN	*0.001*	1.26	1.1	1.45

pM	*< 0.001*	3.51	2.41	5.09

Grade	*0.03*	1.23	1.02	1.48

				

**PFS**				

L1CAM	*0.022*	1.34	1.04	1.73

pT	*< 0.001*	1.57	1.37	1.79

pN	*0.011*	1.2	1.04	1.38

pM	*< 0.001*	5.81	3.82	8.82

Grade	*0.018*	1.25	1.04	1.49

OS overall survival, PFS progression free survival, exp(B) instantaneous relative risk of an event if parameter is present, CI confidence interval

#### L1CAM expression is correlated with EMT markers

In the total cohort L1CAM was positively correlated with slug, both tumoral and stromal vimentin, and inversely with E-cadherin (table 4). L1CAM correlated positively with cytosolic but not membranous or nuclear beta-catenin in tumor cells (table 4). On the whole sections, L1CAM was often found at the tumor-stroma interface (Figure 1B, F and 2A, B) and E-cadherin in the tumor center (Figure 2A-D). Likewise, up-regulated vimentin in tumor cells was often found at the tumor-stroma interface and in areas away from E-cadherin (Figure 2C). Slug was also found at the tumor-stroma interface and inversely correlated with E-cadherin (Figure 2D) which was significant (p-value 0.005, tau = -0.128).

**Table 4:** Correlation of L1CAM with EMT markers

		Slug	Beta-catenin			E-cadherin	Vimentin	
		(nuc)	(mem)	(cyto)	(nuc)	(mem)	(cyto)	(stroma)
L1CAM	p	*< 0.05*	ns	*0.001*	ns	*0.001*	*< 0.001*	*0.001*

(mem)	tau	0.101	-0.073	0.136	0.036	-0.139	0.249	0.137

p p-value, tau correlation coefficient, nuc nucleus, mem membrane, cyto cytoplasm

#### L1CAM expression *in-vitro*

**Figure 4:**
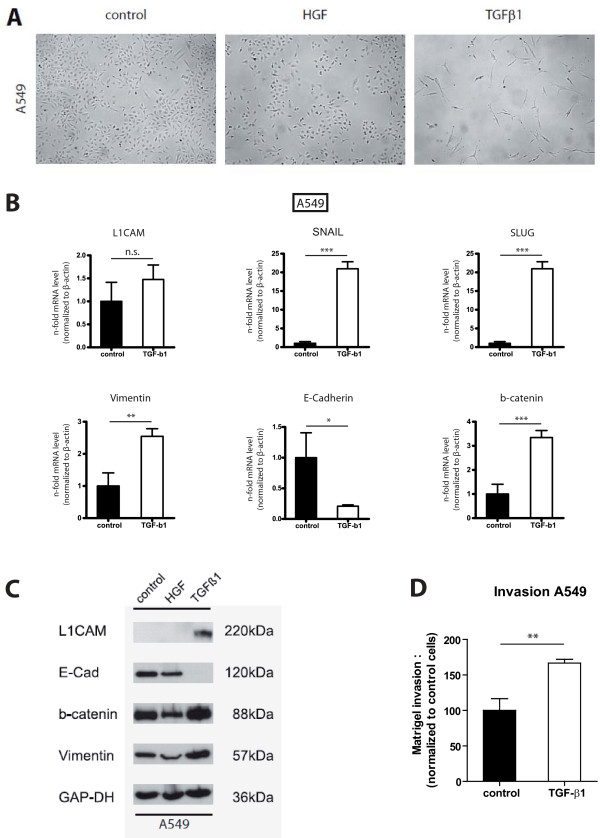
**L1CAM in A549**. A) Stimulation of A549 cells with TGF-beta1 induces a mesenchymal phenotype whereas HGF does not alter cell morphology. Note that TGF-beta1 treatment affected the proliferation of A549 cells. **B/C) **A549 cells stimulated with TGF-beta1 show an EMT phenotype and increased L1CAM expression on mRNA and protein level. One representative experiment of n = 3 is shown. GAP-DH loading control. **D) **Matrigel invasion of A549 cells is enhanced by TGF-beta1 stimulation. The error bars represent mean values ± SEM. One representative experiment of n = 3 is shown.

The correlation of L1CAM expression with invasion in the TMA analysis prompted us to study the pro-invasive function of L1CAM *in-vitro*. A549 cells were treated for 7 days with 5ng/mL HGF or 10ng/mL TGF-beta1 and an EMT like phenotype was induced (Figure 4A). By RT-PCR analysis we found L1CAM, snail, slug, vimentin and beta-catenin mRNA upregulated in A549 cells after TGF-beta1 stimulation for 7 days (Figure 4B). In contrast, E-cadherin mRNA was downregulated in A549 cells (Figure 4B). Similar results were observed for another lung adenocarcinoma cell line H1395 (data not shown). On the protein level, TGF-beta1 induced increased L1CAM, vimentin and beta-catenin expression in A549 cells whereas E-cadherin was downregulated (Figure 4C). In a matrigel invasion assay, TGF-beta1 treatment augmented invasion of A549 cells (Figure 4D).

**Figure 5:**
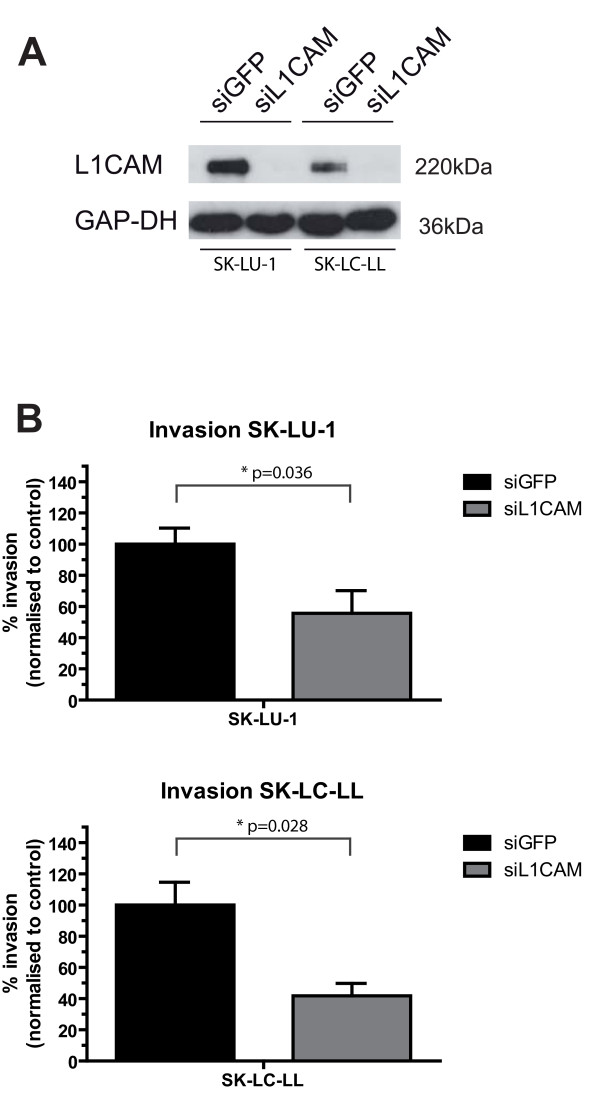
**SiRNA knockdown reduces matrigel invasion**. **A) **SiRNA knockdown suppresses L1CAM expression in SK-LU-1 and SK-LC-LL cells. **B) **L1CAM siRNA knockdown significantly reduces matrigel invasion of SK-LU-1 and SK-LC-LL cells. The error bars represent mean values **± **SEM. One representative experiment of n = 3 is shown.

The cell lines SK-LU-1 and SK-LC-LL neither showed a change in morphology nor L1CAM induction after TGF-beta1 stimulation (data not shown). L1CAM but not GFP siRNA knockdown completely abrogated L1CAM expression in SK-LU-1 and SK-LC-LL cells (Figure 5A) and led to significantly reduced matrigel invasion of both cell lines (SK-LU-1 p = 0.036, SK-LC-LL p = 0.028, Figure 5B). For A549 cells a difference in matrigel invasion was not observed after siRNA-mediated knockdown of L1CAM (data not shown).

### Discussion

In this study, we demonstrate that L1CAM protein is expressed in NSCLC and is correlated with vessel infiltration, metastasis, and a poor prognosis. *In-vivo *data suggests that L1CAM is involved in EMT of lung cancer. Further, we provide evidence that L1CAM is involved in NSCLC cell invasion.

L1CAM expression was found in 25% of NSCLC by immunohistochemistry, using our previously described monoclonal anti-L1CAM antibody (clone 14.10), which is directed to the ectodomain of L1CAM (CD171).

Three hundred fifty-three tumors with two L1CAM negative cores were defined as negative. It cannot be ruled out that some of these cases have L1CAM expression due to intratumoral heterogeneity. However, we found L1CAM expression heterogeneity of 13.5%. In these cases, different staining scores were observed in the two cores of the tumor. Of these, 34 tumors had the score "0 and 1" or "1 and 0". These are less than 10% compared to the 353 cases that are L1CAM negative for both cores. In 10 randomly chosen L1CAM negative cases identified by TMA we did not observe focal L1CAM positivity on whole sections. Our finding of L1CAM expression in non-small cell lung cancer is in contrast to a previous protein expression profile study across several human tissues, which was not able to identify L1CAM expression in NSCLC [24]. This discrepancy is most probably due to the low case number (10 SCC/5 ADCA) in that recent expression analysis [24]. Importantly, other studies identified L1CAM expression in small cell lung carcinoma, in pulmonary carcinoids and in large cell lung carcinomas [10,25]. Interestingly, a relationship to prognosis was also demonstrated in large cell lung carcinomas [25], corroborating our findings in NSCLC. Only L1CAM was of uni- as well as multivariate prognostic significance for OS and PFS among all biomarkers tested. These data are in line with published prognostic data on L1CAM expression in extrahepatic cholangiocarcinoma, gastric, breast, colorectal, ovarian, endometrial, and pancreatic duct carcinoma [6,21,26-30].

We observed a negative correlation of L1CAM with E-cadherin and a positive correlation with slug/vimentin/beta-catenin, indicating a potential role of L1CAM in EMT of NSCLC. Our results are in concordance with findings in colorectal, breast and pancreatic carcinoma where L1CAM expression was clearly involved in the EMT program [9,16,23]. It is unclear how L1CAM up-regulation exactly occurs in NSCLC. Several mechanisms have been described so far: In colorectal carcinoma, aberrant L1CAM expression was attributed to a hyperactive beta-catenin/TCF pathway [23] or to DNA hypomethylation at CpG islands of the L1CAM promoter [31]. In a pancreatic cancer cell line L1CAM expression was regulated via binding of the transcription factor slug to the L1CAM promoter, induced by TGF-beta1 and mediated by c-jun NH2-terminal kinase [9].

In A549 cells, L1CAM expression was inducible by TGF-beta1 which also induced beta-catenin and vimentin and downregulated E-cadherin. We found the EMT transcription factors slug and snail induced by TGF-beta1 on the mRNA level. Our results would favor a model in which aberrant L1CAM expression in NSCLC is mediated by slug and an activated beta-catenin pathway. A knockdown of slug or snail was not performed so that we cannot prove this hypothesis. L1CAM knockdown in the cell lines SK-LU-1 and SK-LC-LL reduced matrigel invasion but we did not observe further up-regulation of L1CAM expression by TGF-beta1 in the latter cell lines.

Recently, EMT has been linked to the cancer stem cell (CSC) phenotype [32], and L1CAM was shown to be co-expressed with the CSC marker CD133 in glioma cells [33]. Molecular targeting of L1CAM in CD133^+ ^glioma cells reduced tumor growth and increased survival *in-vivo *[33]. In a widely accepted hypothesis CSC's are believed to be endowed with increased drug resistance [34]. The existence of bronchoalveolar stem cells (BASC's) and their key role in KRAS-induced lung cancer was proven although it is not assured that BASC's can induce a histophenocopy of the initial tumor in secondary or tertiary hosts (for review see [34]). It remains to be seen if the dismal prognosis that we observe in the L1CAM positive NSCLC subgroup is related to the presence of CSC's.

We observed a correlation of L1CAM protein up-regulation with blood vessel invasion and metastasis in NSCLC. Further, L1CAM was accentuated at the tumor-stroma interface but decreased in central parts, suggesting an L1CAM involvement in tumor cell invasion. SiRNA knockdown of L1CAM conferred a less invasive phenotype in the two cell lines tested, supporting our *in-vivo *observation. On whole sections, we found strong L1CAM expression of tumor cells close to and inside intratumoral blood vessels. Up-regulation of L1CAM at the invasive front was also found in colorectal cancer [23]. NSCLC has a highly desmoplastic stroma similar to pancreatic duct carcinoma, often with formation of a central scar. It is unclear whether this prominent fibrotic reaction is inhibiting or rather promoting tumor cell migration. Therefore the question arises whether up-regulation of L1CAM is a prerequisite for infiltrating tumor cells to reach and invade neo-angiogenetic blood vessels. This hypothesis requires further systematic investigation by *in-vitro *and *in-vivo *vessel invasion assays. Two recent studies showed that a soluble form of L1CAM acts pro-angiogenic and L1CAM expression in the tumor endothelium mediates selective tumor cell transmigration in pancreatic adenocarcinoma [35,36].

Anti-L1CAM therapy altered L1CAM gene expression *in-vitro *as well as reduced tumor growth in a mouse model of intraperitoneally transplanted ovarian cancer cells [37-40]. Thus, anti-L1CAM agents may be used in the setting of intracavitary chemotherapy of malignant effusions. Since protein expression in the carcinoma cells was of similar intensity compared with adjacent peripheral nerves and proximal tubules of the kidney, systemic therapy with mAb to L1CAM could potentially lead to neurological or renal complications.

## Conclusions

We demonstrate that the cell adhesion molecule L1CAM is aberrantly expressed in a subset of NSCLC and independently prognostic for poor survival. Further, we show that L1CAM expression is induced by TGFbeta1 and that siRNA knockdown of L1CAM reduces matrigel invasion. The mechanism by which L1CAM leads to a more aggressive tumor phenotype is potentially related to EMT *in-vivo *and *in-vitro*. A targeted tumoral anti-L1CAM therapy could support anti-epidermal growth factor receptor or anti-vascular endothelial growth factor inhibition in NSCLC.

## Materials and methods

### Patient cohort

Tumor tissue of 472 consecutive patients with surgically resected primary NSCLC between 1993 and 2002 including 244 squamous cell carcinomas (SCC; 51.7%) and 228 adenocarcinomas (ADCA; 48.3%) was used for construction of a tissue microarray (TMA). Briefly, each patient's tumor was represented by two 0.6mm cores. Histotypes like large cell, adeno-squamous, neuroendocrine or sarcomatoid carcinoma, metastases or relapsing tumors and neo-adjuvantly treated patients were not included. Histotypes were entirely reviewed on H&E and mucin stained sections and controlled by IHC according to recent recommendations [41]. Presence of blood vessel infiltration was identified on H&E and Elastica-Van-Gieson stained whole sections. Progression-free (PFS) and overall (OS) survival data was available for 456 patients. The mean follow-up time was 43 (range 0-169, median 25) and 51 months (range 1-169 months, median 41) for PFS and OS, respectively. The study was approved by the ethical committee of the Kanton of Zurich (reference number StV-29-2009).

### Immunohistochemistry and -fluorescence

Immunohistochemistry (IHC) of TMA sections was performed using the following primary antibodies: mAb anti-E-cadherin (Cell Marque Lifescreen Ltd., Rocklin, CA, USA, clone EP700Y, 1:200), mAb anti-beta-catenin (BD Transduction laboratories, Lexington, KY USA, clone 14/beta-catenin, 1:50), mAb anti-vimentin (DAKO A/S, Glostrup, Denmark, 1:250), and mAb anti-slug (Cell Signaling Technology, Inc, Danvers, MA, USA, clone C19G7, 1:100). MAb anti-L1CAM (clone 14.10, directed to the ectodomain, 1:200) was generated as described [24]. Antibodies were tested on a multi tissue TMA for appropriate dilutions. Two protocols were applied: First, on a Ventana Benchmark^® ^platform (Ventana Medical Systems, Tucson, AZ, USA), the CC1 standard pretreatment with 60 min boiling in pH 8 Tris buffer was followed by incubation with primary mAb (E-cadherin, beta-catenin, vimentin) for 60 min at room temperature (RT) and development with the Ultraview-HRP kit, including incubation with respective secondary ab for 30 min at RT. Second, on a Leica Bond^® ^platform (Vision Biosystems, Melbourne, Australia), the H2 standard pre-treatment with 60 min boiling in pH8 Tris buffer was followed by incubation with primary mAb (L1CAM, slug) for 30 min at RT and development with the Refine-DAB Bond kit, including incubation with secondary ab for 30 min at RT and additional polymer amplification. All primary antibodies were diluted in Tris/BSA. To visualize the topographic distribution of protein expression, representative whole sections (n = 5) were stained with double IHC or double IF. For IHC, the Leica Bond^® ^platform with Bond Polymer AP Red Detection and Polymer Refine Detection kits was used. For both systems, hematoxylin counterstain was applied. For IF, sections were boiled for 20 min in citrate buffer and blocked with 5% goat serum for 10 min and incubated overnight with first mAb at 4°C. After washings, slides were probed with an F(ab) goat anti-mouse Alexa-488 (spectrum green, 1:100) or Alexa-546 (spectrum orange, 1:100) for 45 min and counterstained with DAPI. Pictures were taken on an Olympus microscope with CCD camera using the AnalySIS software (Olympus, Munster, Germany).

Intensity of immunoreactivity was semi-quantitatively scored, applying a four-tiered system (0 to 3). L1CAM was independently scored by two pathologists (VT&SH; interobserver kappa-value 0.78). Both scores showed similar results in correlation with EMT markers and clinicopathological parameters. One score (VT) is shown. Peripheral nerves served as internal positive control for L1CAM staining. For L1CAM and E-cadherin membranous staining was counted (score 0 negative, score 1 faint discontinuous, score 2 moderate continuous, score 3 strong continuous). Beta-catenin was separately scored at the plasma membrane, the cytoplasm and the nucleus. Slug was scored in the nucleus. Vimentin was scored in the tumor cell cytoplasm and in peritumoral stromal cells. The two TMA cores were summed up to the sum intensity score (range 0-6).

### Cell culture

The lung carcinoma cell lines A549 (ADCA; obtained from ATCC, #CCL-185), SK-LU-1 (ADCA; obtained from ATCC, #HTB-57), H1395 and SK-LC-LL (ADCA and SCC, respectively; obtained from Dr. Reinhard Schwartz-Albiez, DKFZ Heidelberg) were cultivated in RPMI-1640 medium supplemented with 10% fetal bovine serum and 10 mM Glutamine at 37°C, 5% CO2 and 100% humidity. Cells were periodically tested by PCR for mycoplasma. For induction of EMT cells were cultivated in the presence of HGF (Promokine, Heidelberg, Germany) 5 ng/ml or TGF-beta1 (Promokine, Heidelberg, Germany) 10 ng/ml for 7 days.

### Quantitative real-time PCR

qRT-PCR was performed as described before [21]. Primers for qRT-PCR were designed using the IDT primer quest programme and were produced by MWG (Ebersberg, Germany). Beta-actin was used as an internal standard. The sequences of primers used are available on request.

### Western blot

SDS-PAGE under non-reducing conditions and transfer of proteins to an Immobilon membrane using semi-dry blotting has been described previously [42]. After blocking with 5% skimmed milk in TBS, the blots were developed with the respective primary antibody as described [21], followed by peroxidase-conjugated secondary antibody and ECL (Perkin-Elmer, Rodgau, Germany) detection. The mAbs used for Western blot analysis were described before [21].

### L1CAM knockdown

For siRNA mediated knockdown, either siGFP or siL1CAM were used. Sequences have been published before [43]. Cells were transfected with Oligofectamine (Invitrogen, Karlsruhe, Germany) 24 h before onset of the Matrigel invasion assay.

### Matrigel invasion assay

Tumor cell invasion *in-vitro *was determined in a double-filter assay. Briefly, Matrigel was layered between two filters, including a lower 5-μm pore nitrocellulose and an upper 8-μm pore polycarbonate filter. The lower filter was fixed by a gel that consisted of human fibrinogen (5 mg/mL) and casein (5 mg/mL) dissolved in serum-containing culture medium. 10^5 ^cells were incubated with the filter sandwich for 20 h in 1 mL of medium. The next day, the sandwich was fixed and the filters separated and DAPI stained. Cells attached to the lower filter were counted, and cell invasion was expressed as the ratio of the cell number on the lower filter to the total number of cells in a proliferation control. Five sections per filter were photographed using a Zeiss fluorescence microscope and counted using Image J software.

### Statistics

PFS time was defined as the interval between surgery and disease progression, death or last contact, respectively. Only documented relapses were accepted for warranting progression. Patients with initial metastatic disease were considered to have PFS = 0. OS time was defined as the interval between surgery and death or last contact, respectively. In case of one core loss, data from the remaining core was carried forward. If both cores were not evaluable (4 patients), the case was excluded (n final = 468). Correlations of protein expression intensities with clinico-pathologic parameters were calculated by Kendall's tau b analysis, with PFS and OS by the Kaplan-Meier method using log rank tests. The median value of the sum score was used as cut-off point for dichotomization into a "L1CAM negative" and "L1CAM positive" group. All cases which did not show equal staining intensity for both cores were counted as heterogeneous. Significant univariate prognostic factors were introduced into multivariate analysis applying Cox proportional hazards regression. Statistical analyzes were performed using PASW, version 18.0. P-values < 0.05 were considered significant. Statistical significance of the cell culture experiments was determined by Student's t test. P-values in Figure 4 are indicated as follows: *< 0.05, **< 0.01 ***< 0.001.

## Competing interests

The authors declare that they have no competing interests.

## Authors' contributions

VT designed the study, evaluated immunohistochemical and -fluorescent stainings, prepared and finalized the figures, performed statistical analyses, coordinated the project and wrote the manuscript. MP performed cell experiments, drafted the manuscript, performed statistical analyses and prepared illustrations. SH evaluated immunohistochemical stainings and acquired data. US performed cell experiments and prepared illustrations. AKB evaluated immunohistochemical stainings and acquired data. GK interpreted acquired data, helped to design the study and contributed to the manuscript. MLS drafted the manuscript and contributed to the cell culture data. WW interpreted acquired clinical data and contributed to the manuscript. HM interpreted acquired clinicopathological data and contributed substantially to the manuscript. PA interpreted cell culture data and drafted the manuscript. AS constructed the tissue microarray, designed the study, interpreted the acquired data and drafted the manuscript. All authors read and approved the final manuscript.

## References

[B1] Lindner J Rathjen FG Schachner M L1 mono- and polyclonal antibodies modify cell migration in early postnatal mouse cerebellum Nature 1983 305 427 430 10.1038/305427a0 6621692

[B2] Schafer MK Altevogt P L1CAM malfunction in the nervous system and human carcinomas Cell Mol Life Sci 2010 67 2425 2437 10.1007/s00018-010-0339-1 20237819 PMC11115577

[B3] Moos M Tacke R Scherer H Teplow D Fruh K Schachner M Neural adhesion molecule L1 as a member of the immunoglobulin superfamily with binding domains similar to fibronectin Nature 1988 334 701 703 10.1038/334701a0 3412448

[B4] Meli ML Carrel F Waibel R Amstutz H Crompton N Jaussi R Moch H Schubiger PA Novak-Hofer I Anti-neuroblastoma antibody chCE7 binds to an isoform of L1-CAM present in renal carcinoma cells Int J Cancer 1999 83 401 408 10.1002/(SICI)1097-0215(19991029)83:3<401::AID-IJC17>3.0.CO;2-A 10495434

[B5] Allory Y Matsuoka Y Bazille C Christensen EI Ronco P Debiec H The L1 cell adhesion molecule is induced in renal cancer cells and correlates with metastasis in clear cell carcinomas Clin Cancer Res 2005 11 1190 1197 15709188

[B6] Fogel M Gutwein P Mechtersheimer S Riedle S Stoeck A Smirnov A Edler L Ben-Arie A Huszar M Altevogt P L1 expression as a predictor of progression and survival in patients with uterine and ovarian carcinomas Lancet 2003 362 869 875 10.1016/S0140-6736(03)14342-5 13678974

[B7] Fogel M Mechtersheimer S Huszar M Smirnov A Abu-Dahi A Tilgen W Reichrath J Georg T Altevogt P Gutwein P L1 adhesion molecule (CD 171) in development and progression of human malignant melanoma Cancer Lett 2003 189 237 247 10.1016/S0304-3835(02)00513-X 12490317

[B8] Gavert N Sheffer M Raveh S Spaderna S Shtutman M Brabletz T Barany F Paty P Notterman D Domany E Ben-Ze'ev A Expression of L1-CAM and ADAM10 in human colon cancer cells induces metastasis Cancer Res 2007 67 7703 7712 10.1158/0008-5472.CAN-07-0991 17699774

[B9] Geismann C Morscheck M Koch D Bergmann F Ungefroren H Arlt A Tsao MS Bachem MG Altevogt P Sipos B Up-regulation of L1CAM in pancreatic duct cells is transforming growth factor beta1- and slug-dependent: role in malignant transformation of pancreatic cancer Cancer Res 2009 69 4517 4526 10.1158/0008-5472.CAN-08-3493 19435915

[B10] Miyahara R Tanaka F Nakagawa T Matsuoka K Isii K Wada H Expression of neural cell adhesion molecules (polysialylated form of neural cell adhesion molecule and L1-cell adhesion molecule) on resected small cell lung cancer specimens: in relation to proliferation state J Surg Oncol 2001 77 49 54 10.1002/jso.1065 11344483

[B11] Thies A Schachner M Moll I Berger J Schulze HJ Brunner G Schumacher U Overexpression of the cell adhesion molecule L1 is associated with metastasis in cutaneous malignant melanoma Eur J Cancer 2002 38 1708 1716 10.1016/S0959-8049(02)00105-3 12175686

[B12] Panicker AK Buhusi M Erickson A Maness PF Endocytosis of beta1 integrins is an early event in migration promoted by the cell adhesion molecule L1 Exp Cell Res 2006 312 299 307 16330023 10.1016/j.yexcr.2005.10.031

[B13] Silletti S Yebra M Perez B Cirulli V McMahon M Montgomery AM Extracellular signal-regulated kinase (ERK)-dependent gene expression contributes to L1 cell adhesion molecule-dependent motility and invasion J Biol Chem 2004 279 28880 28888 10.1074/jbc.M404075200 15128735

[B14] Kiefel H Bondong S Erbe-Hoffmann N Hazin J Riedle S Wolf J Pfeifer M Arlt A Schafer H Muerkoster SS Altevogt P L1CAM-integrin interaction induces constitutive NF-kappaB activation in pancreatic adenocarcinoma cells by enhancing IL-1beta expression Oncogene 2010 29 4766 4778 10.1038/onc.2010.230 20543863

[B15] Soltermann A Tischler V Arbogast S Braun J Probst-Hensch N Weder W Moch H Kristiansen G Prognostic significance of epithelial-mesenchymal and mesenchymal-epithelial transition protein expression in non-small cell lung cancer Clin Cancer Res 2008 14 7430 7437 10.1158/1078-0432.CCR-08-0935 19010860

[B16] Shtutman M Levina E Ohouo P Baig M Roninson IB Cell adhesion molecule L1 disrupts E-cadherin-containing adherens junctions and increases scattering and motility of MCF7 breast carcinoma cells Cancer Res 2006 66 11370 11380 10.1158/0008-5472.CAN-06-2106 17145883

[B17] Thiery JP Epithelial-mesenchymal transitions in tumour progression Nat Rev Cancer 2002 2 442 454 10.1038/nrc822 12189386

[B18] Mimeault M Batra SK Interplay of distinct growth factors during epithelial mesenchymal transition of cancer progenitor cells and molecular targeting as novel cancer therapies Ann Oncol 2007 18 1605 1619 10.1093/annonc/mdm070 17355951

[B19] Heuberger J Birchmeier W Interplay of cadherin-mediated cell adhesion and canonical Wnt signaling Cold Spring Harb Perspect Biol 2010 2 a002915 10.1101/cshperspect.a002915 20182623 PMC2828280

[B20] Kiefel H Pfeifer M Bondong S Hazin J Altevogt P Linking L1CAM-mediated signaling to NF-kappaB activation Trends Mol Med 2011 17 178 187 10.1016/j.molmed.2010.11.005 21195665

[B21] Huszar M Pfeifer M Schirmer U Kiefel H Konecny GE Ben-Arie A Edler L Munch M Muller-Holzner E Jerabek-Klestil S Up-regulation of L1CAM is linked to loss of hormone receptors and E-cadherin in aggressive subtypes of endometrial carcinomas J Pathol 2010 220 551 561 10.1002/path.2673 20077528

[B22] Hajra KM Chen DY Fearon ER The SLUG zinc-finger protein represses E-cadherin in breast cancer Cancer Res 2002 62 1613 1618 11912130

[B23] Gavert N Conacci-Sorrell M Gast D Schneider A Altevogt P Brabletz T Ben-Ze'ev A L1, a novel target of beta-catenin signaling, transforms cells and is expressed at the invasive front of colon cancers J Cell Biol 2005 168 633 642 10.1083/jcb.200408051 15716380 PMC2171754

[B24] Huszar M Moldenhauer G Gschwend V Ben-Arie A Altevogt P Fogel M Expression profile analysis in multiple human tumors identifies L1 (CD171) as a molecular marker for differential diagnosis and targeted therapy Hum Pathol 2006 37 1000 1008 10.1016/j.humpath.2006.03.014 16867862

[B25] Kim HS Yi SY Jun HJ Ahn JS Ahn MJ Lee J Kim Y Cui ZY Hong HJ Kim JM L1 cell adhesion molecule as a predictor for recurrence in pulmonary carcinoids and large-cell neuroendocrine tumors APMIS 2009 117 140 146 10.1111/j.1600-0463.2009.02433.x 19239436

[B26] Boo YJ Park JM Kim J Chae YS Min BW Um JW Moon HY L1 expression as a marker for poor prognosis, tumor progression, and short survival in patients with colorectal cancer Ann Surg Oncol 2007 14 1703 1711 10.1245/s10434-006-9281-8 17211730

[B27] Kodera Y Nakanishi H Ito S Misawa K Ito Y Nakayama G Koike M Fujiwara M Yamamura Y Nakao A Expression of L1 cell adhesion molecule is a significant prognostic factor in pT3-stage gastric cancer Anticancer Res 2009 29 4033 4039 19846947

[B28] Schroder C Schumacher U Fogel M Feuerhake F Muller V Wirtz RM Altevogt P Krenkel S Janicke F Milde-Langosch K Expression and prognostic value of L1-CAM in breast cancer Oncol Rep 2009 22 1109 1117 19787228 10.3892/or_00000543

[B29] Li S Jo YS Lee JH Min JK Lee ES Park T Kim JM Hong HJ L1 cell adhesion molecule is a novel independent poor prognostic factor of extrahepatic cholangiocarcinoma Clin Cancer Res 2009 15 7345 7351 10.1158/1078-0432.CCR-09-0959 19920102

[B30] Ben QW Wang JC Liu J Zhu Y Yuan F Yao WY Yuan YZ Positive expression of L1-CAM is associated with perineural invasion and poor outcome in pancreatic ductal adenocarcinoma Ann Surg Oncol 2010 17 2213 2221 10.1245/s10434-010-0955-x 20162456

[B31] Kato K Maesawa C Itabashi T Fujisawa K Otsuka K Kanno S Tada H Tatemichi Y Kotani K Oikawa H DNA hypomethylation at the CpG island is involved in aberrant expression of the L1 cell adhesion molecule gene in colorectal cancer Int J Oncol 2009 35 467 476 19639167 10.3892/ijo_00000358

[B32] Mani SA Guo W Liao MJ Eaton EN Ayyanan A Zhou AY Brooks M Reinhard F Zhang CC Shipitsin M The epithelial-mesenchymal transition generates cells with properties of stem cells Cell 2008 133 704 715 10.1016/j.cell.2008.03.027 18485877 PMC2728032

[B33] Bao S Wu Q Li Z Sathornsumetee S Wang H McLendon RE Hjelmeland AB Rich JN Targeting cancer stem cells through L1CAM suppresses glioma growth Cancer Res 2008 68 6043 6048 10.1158/0008-5472.CAN-08-1079 18676824 PMC2739001

[B34] Eramo A Haas TL De Maria R Lung cancer stem cells: tools and targets to fight lung cancer Oncogene 2010 29 4625 4635 10.1038/onc.2010.207 20531299

[B35] Friedli A Fischer E Novak-Hofer I Cohrs S Ballmer-Hofer K Schubiger PA Schibli R Grunberg J The soluble form of the cancer-associated L1 cell adhesion molecule is a pro-angiogenic factor Int J Biochem Cell Biol 2009 41 1572 1580 10.1016/j.biocel.2009.01.006 19401151

[B36] Issa Y Nummer D Seibel T Muerkoster SS Koch M Schmitz-Winnenthal FH Galindo L Weitz J Beckhove P Altevogt P Enhanced L1CAM expression on pancreatic tumor endothelium mediates selective tumor cell transmigration J Mol Med 2009 87 99 112 10.1007/s00109-008-0410-7 18931829

[B37] Arlt MJ Novak-Hofer I Gast D Gschwend V Moldenhauer G Grunberg J Honer M Schubiger PA Altevogt P Kruger A Efficient inhibition of intra-peritoneal tumor growth and dissemination of human ovarian carcinoma cells in nude mice by anti-L1-cell adhesion molecule monoclonal antibody treatment Cancer Res 2006 66 936 943 10.1158/0008-5472.CAN-05-1818 16424028

[B38] Knogler K Grunberg J Zimmermann K Cohrs S Honer M Ametamey S Altevogt P Fogel M Schubiger PA Novak-Hofer I Copper-67 radioimmunotherapy and growth inhibition by anti-L1-cell adhesion molecule monoclonal antibodies in a therapy model of ovarian cancer metastasis Clin Cancer Res 2007 13 603 611 10.1158/1078-0432.CCR-06-1486 17255283

[B39] Gast D Riedle S Issa Y Pfeifer M Beckhove P Sanderson MP Arlt M Moldenhauer G Fogel M Kruger A Altevogt P The cytoplasmic part of L1-CAM controls growth and gene expression in human tumors that is reversed by therapeutic antibodies Oncogene 2008 27 1281 1289 10.1038/sj.onc.1210747 17952127

[B40] Wolterink S Moldenhauer G Fogel M Kiefel H Pfeifer M Luttgau S Gouveia R Costa J Endell J Moebius U Altevogt P Therapeutic antibodies to human L1CAM: functional characterization and application in a mouse model for ovarian carcinoma Cancer Res 2010 70 2504 2515 10.1158/0008-5472.CAN-09-3730 20215505

[B41] Ring BZ Seitz RS Beck RA Shasteen WJ Soltermann A Arbogast S Robert F Schreeder MT Ross DT A novel five-antibody immunohistochemical test for subclassification of lung carcinoma Mod Pathol 2009 22 1032 1043 10.1038/modpathol.2009.60 19430419

[B42] Gutwein P Oleszewski M Mechtersheimer S Agmon-Levin N Krauss K Altevogt P Role of Src kinases in the ADAM-mediated release of L1 adhesion molecule from human tumor cells J Biol Chem 2000 275 15490 15497 10.1074/jbc.275.20.15490 10809781

[B43] Kiefel H Bondong S Erbe-Hoffmann N Hazin J Riedle S Wolf J Pfeifer M Arlt A Schafer H Muerkoster SS Altevogt P L1CAM-integrin interaction induces constitutive NF-kappaB activation in pancreatic adenocarcinoma cells by enhancing IL-1beta expression Oncogene 2010 10.1038/onc.2010.230 20543863

